# Catalytic Fields as a Tool to Analyze Enzyme Reaction Mechanism Variants and Reaction Steps

**DOI:** 10.1021/acs.jpcb.1c05256

**Published:** 2021-10-14

**Authors:** Paweł Kędzierski, Martyna Moskal, W. Andrzej Sokalski

**Affiliations:** Department of Chemistry, Wrocław University of Science and Technology, Wyb. Wyspiańskiego 27, 50-370 Wrocław, Poland

## Abstract

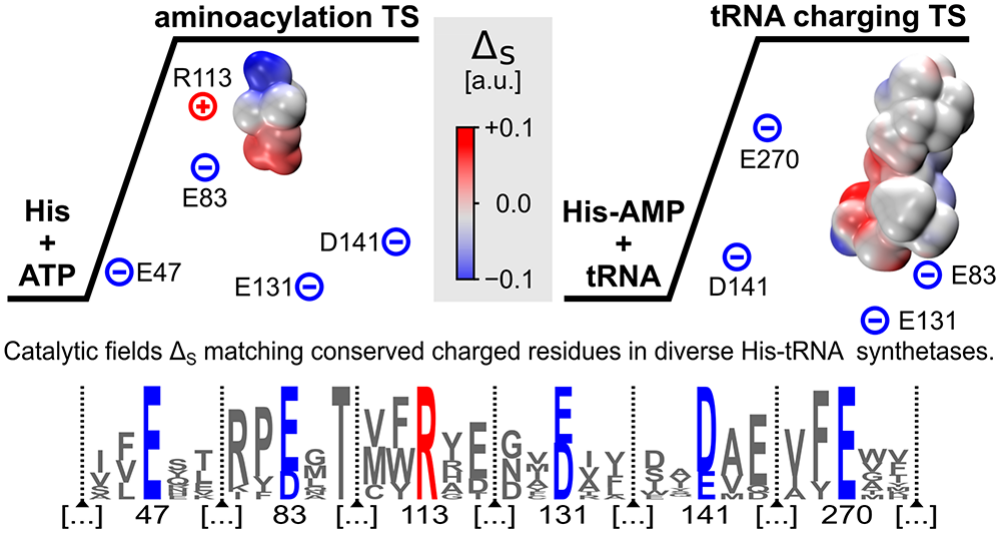

Catalytic fields representing the topology of the optimal molecular environment charge distribution that reduces the activation barrier have been used to examine alternative reaction variants and to determine the role of conserved catalytic residues for two consecutive reactions catalyzed by the same enzyme. Until now, most experimental and conventional top-down theoretical studies employing QM/MM or ONIOM methods have focused on the role of enzyme electric fields acting on broken bonds of reactants. In contrast, our bottom-up approach dealing with a small reactant and transition-state model allows the analysis of the opposite effects: how the catalytic field resulting from the charge redistribution during the enzyme reaction acts on conserved amino acid residues and contributes to the reduction of the activation barrier. This approach has been applied to the family of histidyl tRNA synthetases involved in the translation of the genetic code into the protein amino acid sequence. Activation energy changes related to conserved charged amino acid residues for 12 histidyl tRNA synthetases from different biological species allowed to compare on equal footing the catalytic residues involved in ATP aminoacylation and tRNA charging reactions and to analyze different reaction mechanisms proposed in the literature. A scan of the library of atomic multipoles for amino acid side-chain rotamers within the catalytic field pointed out the change in the Glu83 conformation as the critical catalytic effect, providing, at low computational cost, insight into the electrostatic preorganization of the enzyme catalytic site at a level of detail that has not yet been accessible in conventional experimental or theoretical methods. This opens the way for rational reverse biocatalyst design at a very limited computational cost without resorting to empirical methods.

## Introduction

Detailed knowledge of enzyme reaction mechanisms, the role of amino acid residues essential for catalytic activity, and the structure of corresponding transition states at atomic resolution could provide invaluable information on the process of the rational catalyst^1,2^ or drug^3^ design, as many enzyme inhibitors are transition-state analogs. However, the available experimental structural data for enzymes typically determined in X-ray diffraction studies are most frequently of low resolution and lack accurate hydrogen atom positions.^4^ They are usually the starting point for tedious trial-and-error searches to locate stationary points on the vast multidimensional energy hypersurface using hybrid quantum mechanics/molecular mechanics (QM/MM)^5^ or ONIOM (our own *n*-layered integrated molecular orbital and molecular mechanics)^6^ techniques.

In this Article, we present an alternative way to analyze enzyme reaction variants based on perturbational intermolecular interaction theory, allowing the partitioning of activation energy changes into well-defined physical components, the analytical representation of which could be the subject of gradual approximations leading to simpler nonempirical models applicable to large molecular systems. Our simple approach could be applied to the preliminary screening of possible mechanisms and the evaluation of the role of charged residues in catalysis in the absence of experimental kinetics data for enzyme mutants. In the case in which such data were available, more comprehensive methods integrating structural and kinetic data could be applied.^7^

Warshel postulated the dominant role of electrostatic interactions in enzyme catalysis based on the empirical valence bond approach,^8^ which was later confirmed by various nonempirical methods. For example, the decomposition of the interaction energies using the hybrid variation-perturbation theory illustrate that short-range nonelectrostatic components could cancel each other to a significant degree and that the electrostatic contribution to the lowering of the activation barrier correlates well (*R* = 0.85) with rigorous MP2 results for chorismate mutase residues.^9^ (See Table 1 in ref (9).) This results from the short-range nature of other major components of the interaction energy, like the exchange decaying exponentially with distance and their canceling each other to a significant degree, leaving the electrostatic energy as the dominant and only additive term, which allows partitioning of the entire system into individual residues. The electrostatic nature of the catalytic activity in various enzymes was independently confirmed later by a nonempirical analysis of electric fields obtained from proton quantum dynamics,^10^ transition path sampling,^11^ full quantum-mechanical electric field,^12^ and electron density^13^ calculations and QM/MM results for proton-coupled electron transfer reactions.^14^ The dominant role of electric fields in catalysis has been summarized in recent reviews.^15,16^ The electrostatic nature of catalytic activity has been recently confirmed by experimental^17^ and advanced theoretical studies,^10−14,18−20^ demonstrating how external electric fields generated by enzyme residues act on bonds broken in reactants. However, in this work, we study the inverse effect, that is, how the catalytic field resulting from charge redistribution during an enzyme reaction would maximize interactions with specific amino acid residues. This allows us to determine, at low computational cost, the optimal amino acid side-chain conformations exerting extreme catalytic activity, constituting a preorganized environment. To evaluate various alternative reaction mechanisms, we employ in this study catalytic fields (CFs) defining the charge distribution of the optimal catalytic environment.^9,21^ The CFs derived from the corresponding transition-state and substrate wave functions have been used here to locate amino acid residues exerting the highest catalytic activity for both enzyme reaction steps and all proposed alternative mechanism variants in histidyl-tRNA synthetase (HisRS).^22,23^ HisRS has been selected on purpose because it represents one of the oldest enzyme families with little similarity among various biological species, except for a set of conserved charged residues, which will be matched here with different CF variants.

Every amino acid tRNA (transfer ribonucleic acid) synthetase (aaRS) catalyzes two consecutive reactions: the aminoacylation of adenosine triphosphate (ATP) and then the transfer of the residue from amino acid-AMP (adenosine monophosphate) to tRNA. Once the most probable mechanism is determined for each half-reaction, it would be possible to analyze the specific roles of conserved residues for each step, or, vice versa, the observed conservative catalytic residues could be used to determine the most probable reaction mechanism. Aminoacyl tRNA synthetases, which are involved in the translation of a DNA nucleotide to a protein amino acid sequence, are divided into two classes possibly originating from different ancestors.^24^ Because of their evolutional age and despite catalyzing essentially the same reaction, aaRSs originating from different organisms possess little sequence similarity, even within each of more than 20 aaRS types, like HisRS. Therefore, the topologies of conserved residues could be regarded as their only common denominator, and they constitute an excellent case to confront this with CFs corresponding to various alternative reaction mechanisms catalyzed by aaRSs. Because of the almost one order of magnitude stronger interactions of charged molecules versus neutral molecule, only conserved charged residues have been considered in this study. To keep our model general for the entire HisRS family, we did not consider the nonconserved residues in this work.

**Figure 1:**
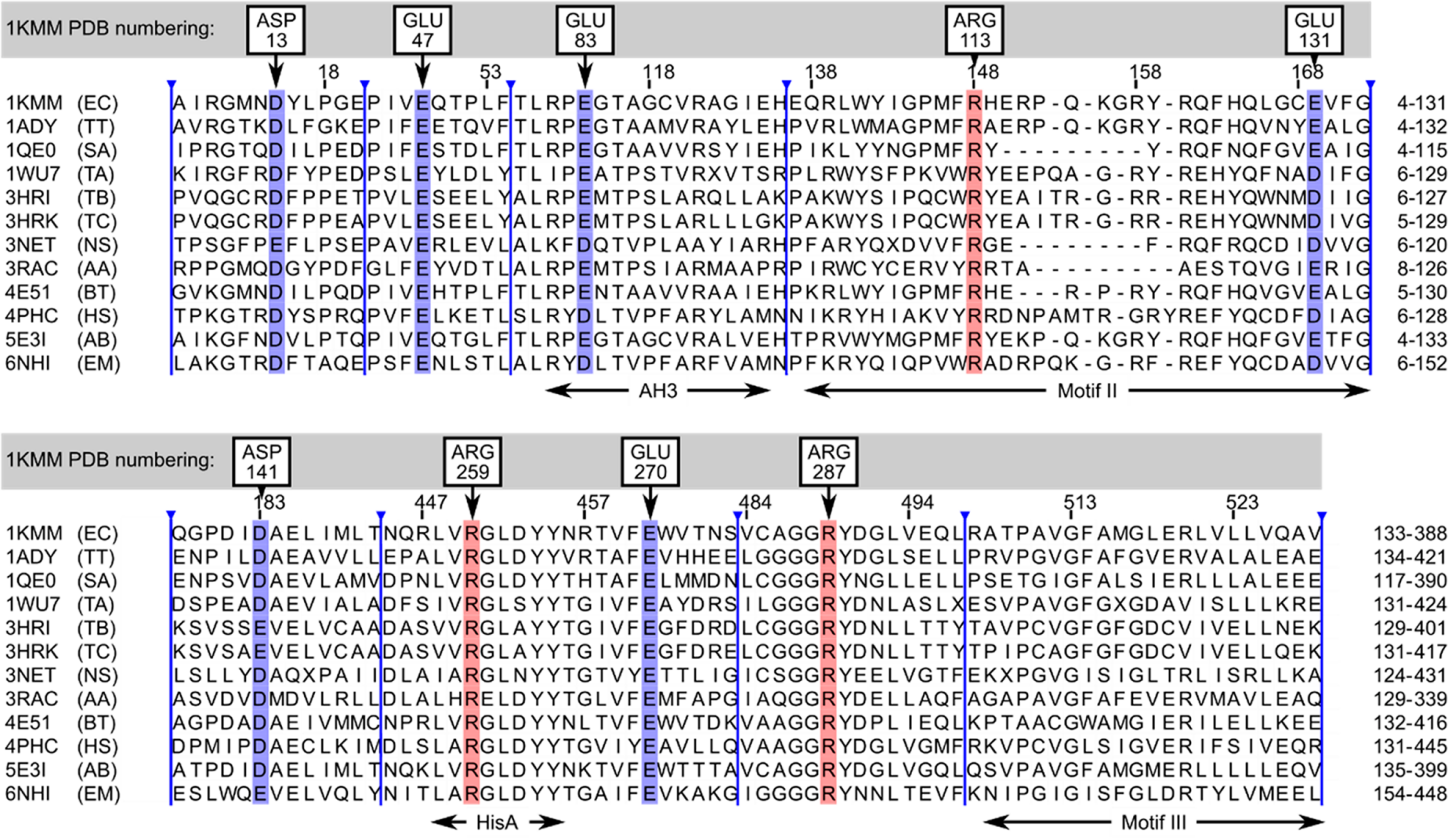
Partial sequence comparison based on the structure alignment of HisRSs originating from 12 different biological organisms. Only the neighborhoods of conservative charged residues and known class II aaRS motifs are presented for brevity. Hidden parts are marked with vertical blue lines.

The first goal of this study is to determine the spatial distribution of conserved charged amino acid residues in class II histidyl tRNA synthetases, representing a wide set of 12 different biological species. The topology of such residues will be confronted with CFs related to both reaction stages 1 to 2 and alternative reaction mechanisms discussed in the literature.^22,23^ Catalytic fields for each reaction variant involving HisRS will be generated to examine activation barrier changes resulting from the presence of conserved charged residues. This would help to point out the most probable mechanisms among several proposed mechanisms. Another goal is to compare the CFs for both reactions, as shown in Figure 1, and proceeding in the HisRS active site to obtain more general knowledge of the catalytic role of conserved residues for each separate reaction step. In addition, the application of the MULTISCAN procedure^21^ will deliver optimal conformations of charged amino acid side chains interacting with each other, permitting the inspection of features of the electrostatic active-site preorganization that are otherwise not easily available from conventional experimental or theoretical techniques.

## Methods

The catalytic activity could be expressed as the difference in the transition state (TS) and substrate (RS) interaction energies, Δ*E*, with the catalytic environment (C) constituting the differential transition state stabilization (DTSS) energy approximating the lowering of the activation barrier.^9,21^
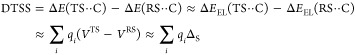
1The final part of eq 1 indicate, that in the extreme approximation, the DTSS contributions could be estimated with point charges representing, for example, formal charges of amino acid side chains interacting with the differential electrostatic potential calculated from the quantum models of the reactants and the transition state, represented by Δ_S_. However, in this work, we rely on a much more precise approximation of the electrostatic term of interaction energies, including all side-chain atoms represented by atomic multipole moments up to the hexadecapole using eq 2. A detailed analysis of all of the interaction energy constituents for enzymes^9,21^ indicated the dominant role of electrostatic term, *E*_EL_, which can be further approximated by cumulative atomic multipole moment (CAMM) expansion^25^
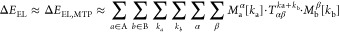
2where and are α and β components of atom-centered multipole tensors of rank *k*_a_ and *k*_b_ for interacting molecules A and B, respectively, and  is the αβ element of the Cartesian interaction tensor containing the partial derivatives of |*R*_ab_|^–1^ of rank *k*_a_ + *k*_b_.

The DTSS could be alternatively estimated (eq 1) as the sum of the products of catalyst atomic charges, *q*_*i*_, and the difference in the molecular electrostatic potentials of the transition state, *V*^TS^, and the substrate, *V*^RS^. The static CF Δ_s_ (eq 3) corresponds to the lowering of the activation barrier by a unit point charge +1 located at any point *i*.
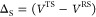
3

The highest catalytic activity is achieved when charged elements of the molecular environment coincide with extreme −Δ_S_ values. Because of the additive nature of electrostatic interactions, −Δ_S_ represents an inverse solution to the optimal catalyst problem. This constitutes a bottom-up approach to catalyst design based on the charge redistribution of reactants at a given reaction step.^21^ It is fundamentally different from conventional top-down methods requiring consideration of the entire protein and numerous assumptions related to the QM/MM boundaries, the selection of protonation states, and the use of empirical force fields.

The activation energy decrease resulting from the presence of specific amino acid residues could be estimated from the eq 1, where molecular electrostatic potentials, *V*, could be obtained either as expectation values from corresponding quantum-chemical wave functions or from a much more compact CAMM representation.^25^ Such distributed moments constitute a natural extension of Mulliken’s population analysis, and the inclusion of higher moments reduces the basis set dependency of atomic charges^25^ and considerably improves the description of the significant anisotropy of charge distribution at the atomic level for reacting systems, where bonds are broken or formed. A more extensive review of the multipolar electrostatics, the errors associated with the use of atomic charges, and its use in force fields can be found in the Popelier review.^26^ In the case of CAMM expansion (eq 2) truncated at the R^–5^ term, the typical error at the equilibrium distance amounts to 5%. It has to be mentioned that there are several nonempirical force-field techniques based on atomic multipole expansion like AMOEBA^27^ or sum of interactions between fragments ab initio (SIBFA).^28^

The use of CAMM atomic multipole expansion was already sufficient to reproduce relative catalytic activities in a series of ketosteroid isomerase^29^ and Kemp eliminase^21^ mutants. Because of the known flexibility of charged amino acid side chains upon ligand binding,^30−32^ corresponding rotamers^33,34^ have been scanned for all conserved residues considering the mutual interactions of charged side chains within the MULTISCAN procedure, previously described in detail.^21^ This allowed us to determine the optimal catalytic amino acid side-chain orientation in the respective CF, which, until recently, has escaped experimental or theoretical observation in conventional studies.^21,32,35^

The calculations included the following steps:

(1) Reaction models were adapted from coordinates published in ref (22) for the aminoacylation step and from ref (23) for the His-tRNA charging step. The aminoacylation reactants and transition state (TS0) were originally optimized using the hybrid ONIOM HF/6-31G**:PM3 theory level.^22^ This model was stripped down to the essential subset of atoms, as presented in thicker representation in Figure 3. Hydrogen atoms were added in place of cut bonds using PyMOL (The PyMOL Molecular Graphics System, version 1.7, Schrödinger). PyMOL was also used to dock the models in the enzyme active site, employing the superposition of their corresponding non-hydrogen atoms onto their counterparts in the His-AMP ligand in the 1KMM Protein Data Bank (PDB) structure. The CAMM electrostatic charge distribution for this step was evaluated for up to rank 4 moments (hexadecapoles) at the HF/6-31G* (5d) level using the GAMESS program (2010-10-01 release or any later version) using the option “$ELMOM IAMM=4 CUM=.T. $END”, generating CAMMs up to hexadecapole. The models of reactants and transition states for the second step of charging the tRNA with the histidyl moiety were originally optimized in the gas phase at the B3LYP/6-31G** level,^23^ including five amino acid residues (Glu83, Arg113, Gln127, Arg259, and Glu270) from the active site. In this work, these models were stripped down to the essential part of the reactants, as shown by thicker representations in Figure 4 for the transition states, capped with hydrogen atoms, and docked with PyMOL, as described above. CAMM was evaluated for them at the HF/6-31G** (5d) level up to hexadecapole moments.

(2) The Python MULTISCAN procedure^21^ calculating multipole electrostatic interaction energies between reactants and amino acid residues, Δ*E*_EL,MTP_, according to eq 2 was used to automatically scan all possible orientations of side chains for the selected set of nearby located conserved charged amino acids in the CF in the search of the lowest DTSS, that is, the minimal activation energy for the considered amino acid set. The CAMM approximation of the interaction energies was exponent-truncated at rank 4.

(3) Atomic multipole values for the amino acid side-chain configuration were interpolated using the CAMM database of amino acid side-chain rotamers calculated at the HF/6-31G* level, available at http://camm.pwr.edu.pl/CAMM.^34^

## Results and Discussion

### Multiple Sequence Alignment of the HisRS Family

The crucial long-range electrostatic interactions of HisRS reactants are with charged amino acids. To determine the location of such conserved HisRS charged residues, we have performed multistructure alignment using 3DCOMB^36^ for 12 histidyl tRNA synthetases from different biological species (see the Supporting Information), for which sufficiently accurate and complete structures were available in the PDB. Among multiple PDB structures of the same protein, only those with the best resolution and a docked ligand with the phosphate group (preferably transition state analog) have been considered, unless a ligand-less structure was the only one available.

**Figure 2:**
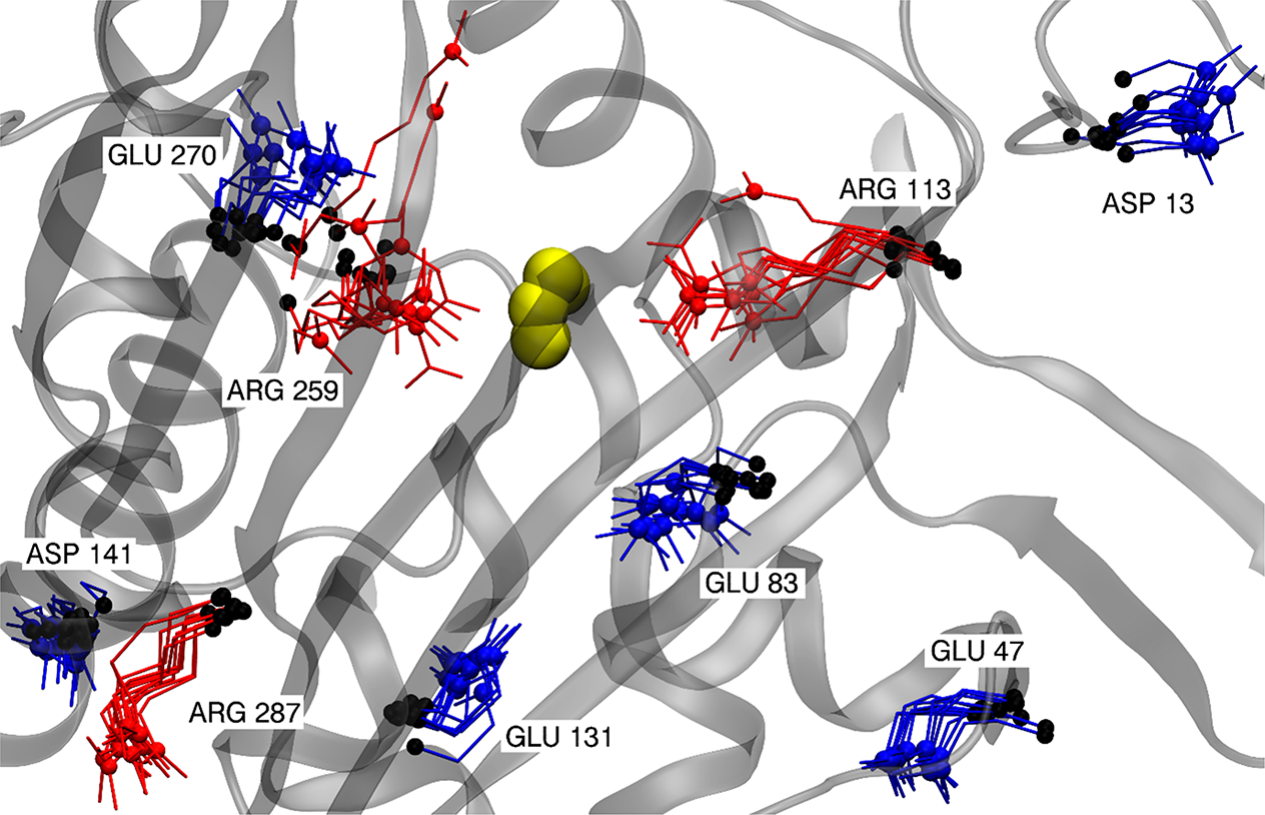
Conserved charged residues for 12 members of the HisRS family superimposed on the 1KMM chain C using the corresponding C_α_ carbon atoms of 9 conserved charged residues, shown as black balls. Blue or red balls indicate terminal carbon atoms C_t_ of negatively or positively charged side chains, respectively. Golden spheres indicate the positions of phosphorus atoms in ligands, if present.

To obtain a more general picture valid for the entire family of HisRSs, our study is limited to residues where the charge is conserved in 100% of HisRSs. The results illustrated in Figure 1 indicate nine charged residues conserved in all considered HisRSs, that is, Asp13, Glu47, Glu83, Arg113, Glu131, Asp141, Arg259, Glu270, and Arg287. (The numbering corresponds to PDB 1KMN and 1KMM structures.) This extends the previous analysis,^37^ where only three bacteria species were considered. More details are presented in the Supporting Information. Figure 2 illustrates the topology of the conserved residues of all 12 structures superimposed using the C_α_ carbon coordinates of conserved residues. To estimate the flexibility of the side chains of these residues, we have compared the root-mean-square deviations (rmsd’s) of C_α_ carbon coordinates with the rmsd’s of C_t_ calculated between corresponding coordinates of carbon atoms representing the side-chain terminal charged guanidinium and carboxyl groups. The C_t_ rmsd’s are always larger than the rmsd’s of C_α_ atoms, in some instances by even more than three times. (See the Supporting Information.) This indicates the significant flexibility of charged side chains already reported in other related studies,^30−32^ which will also be extensively explored in this work.

Information about conserved residues has sometimes been helpful in the search for enzyme reaction mechanisms;^38,39^ however, to our knowledge, there has not yet been an attempt in the literature to verify alternative enzyme reaction mechanism variants by the systematic matching of all conserved amino acids with CFs.

### Stationary Points for Reactions Catalyzed by HisRS

**Scheme 1:**
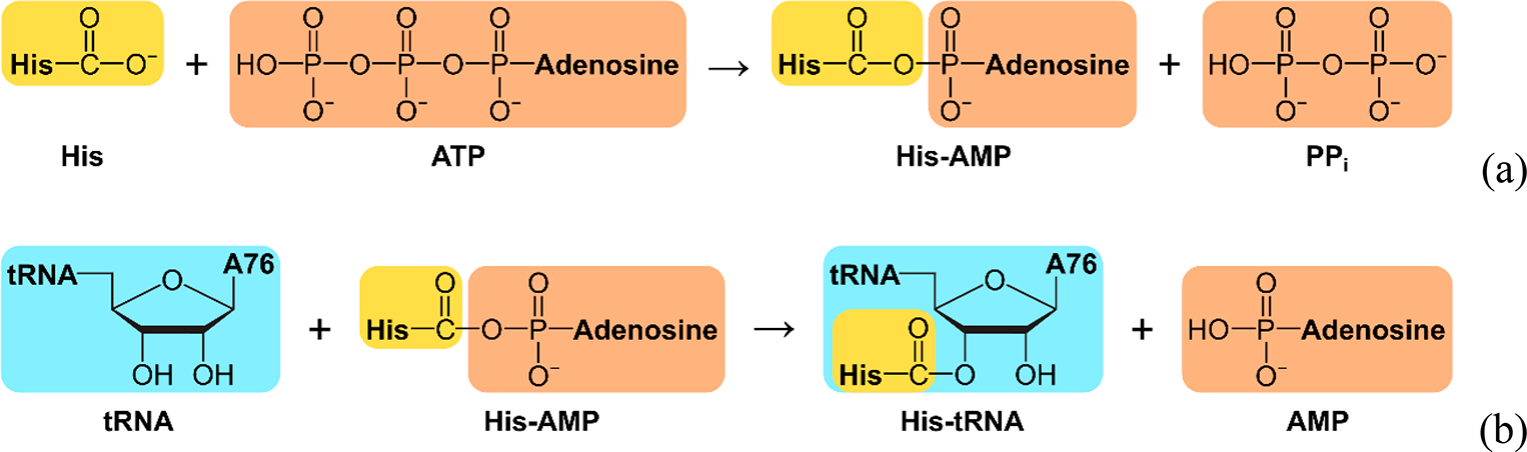
Schematic Representation of Both Consecutive Reactions Considered in This Study PP_i_ denotes pyrophosphate, and A76 denotes the adenine of the tRNA terminal nucleotide.

In the first part, we analyze the CF for the ATP aminoacylation reaction mechanism (Scheme 1a) proposed by Banik and Nandi.^22^ For the second reaction (Scheme 1b) catalyzed by HisRS, three possible mechanism variants of transfer of the aminoacyl moiety to tRNA have been proposed by Liu and Gauld.^23^

### Analysis of Catalytic Activity

**Figure 3:**
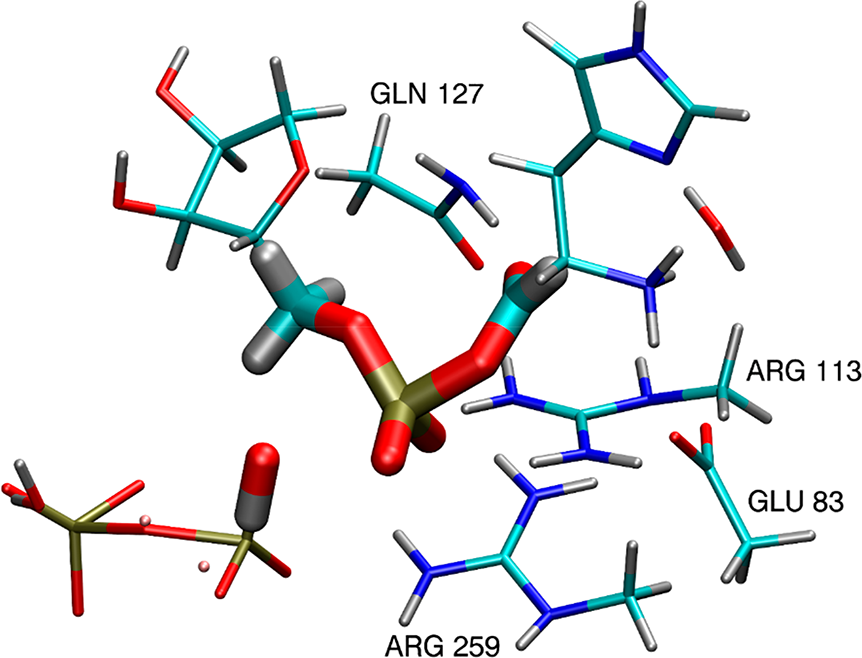
Transition state TS0 for the ATP aminoacylation reaction mechanism (Scheme 1a), as proposed by Banik and Nandi,^22^ where the thicker representation corresponds to the smaller model used to obtain the corresponding catalytic field Δ_S_.

**Figure 4:**
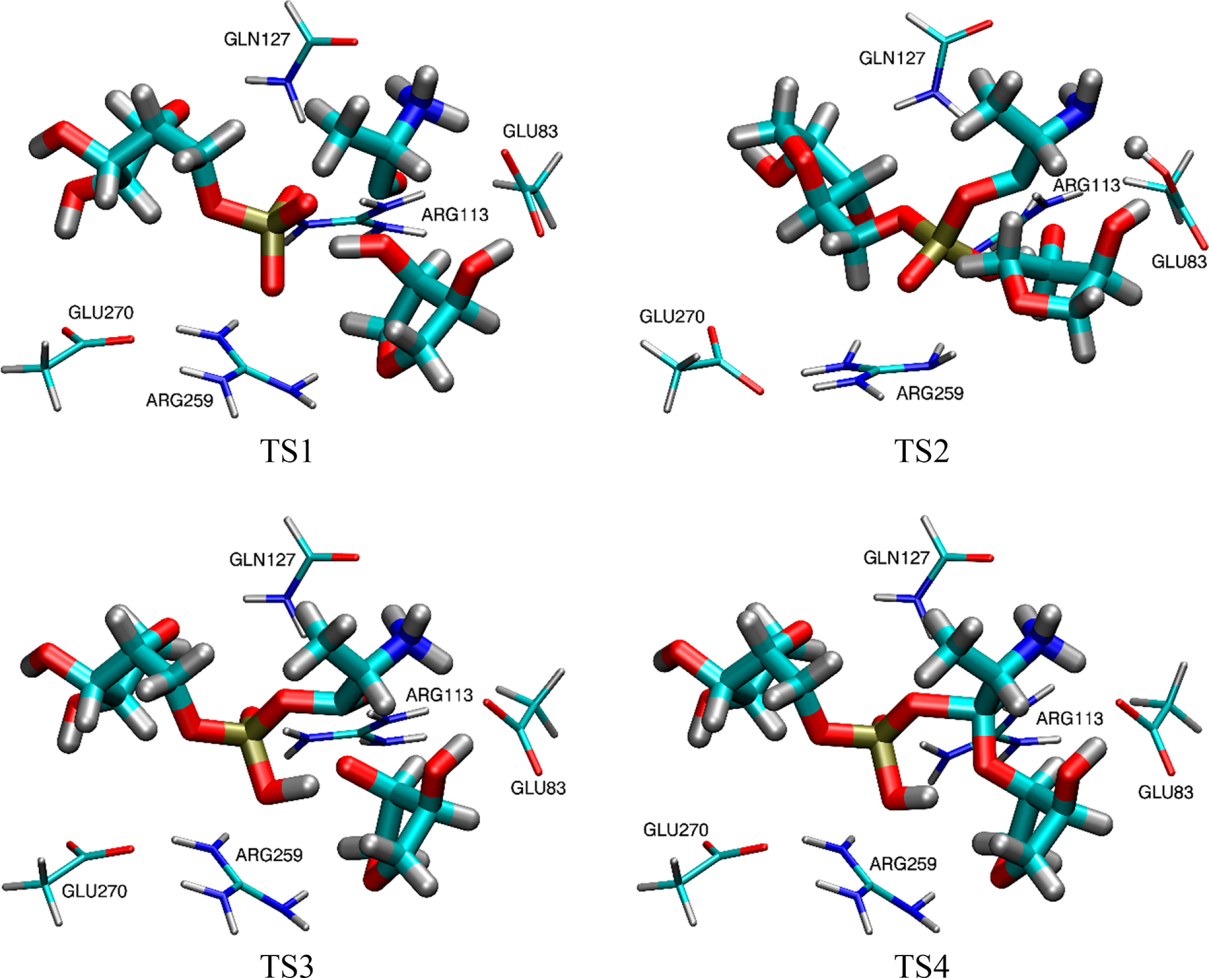
Transition states TS1, TS2, TS3, and TS4 for the His-AMP tRNA charging reaction mechanisms (Scheme 1b), as proposed by Liu and Gauld,^23^ where the thicker lines correspond to the smaller model used here to obtain the corresponding catalytic field Δ_S_.

To estimate changes in the activation barrier DTSS due to every conserved residue for each reaction step or variant, we have generated CFs using the CAMM derived directly from wave functions of the transition state and substrate smaller models illustrated in Figures 3 and 4. Such simple models are composed of the CAMM describing the charge redistribution on the reaction path from the substrate to the transition state without including any amino acid residue present in the higher level part of the ONIOM or QM/MM original calculations.^22,23^

**Figure 5:**
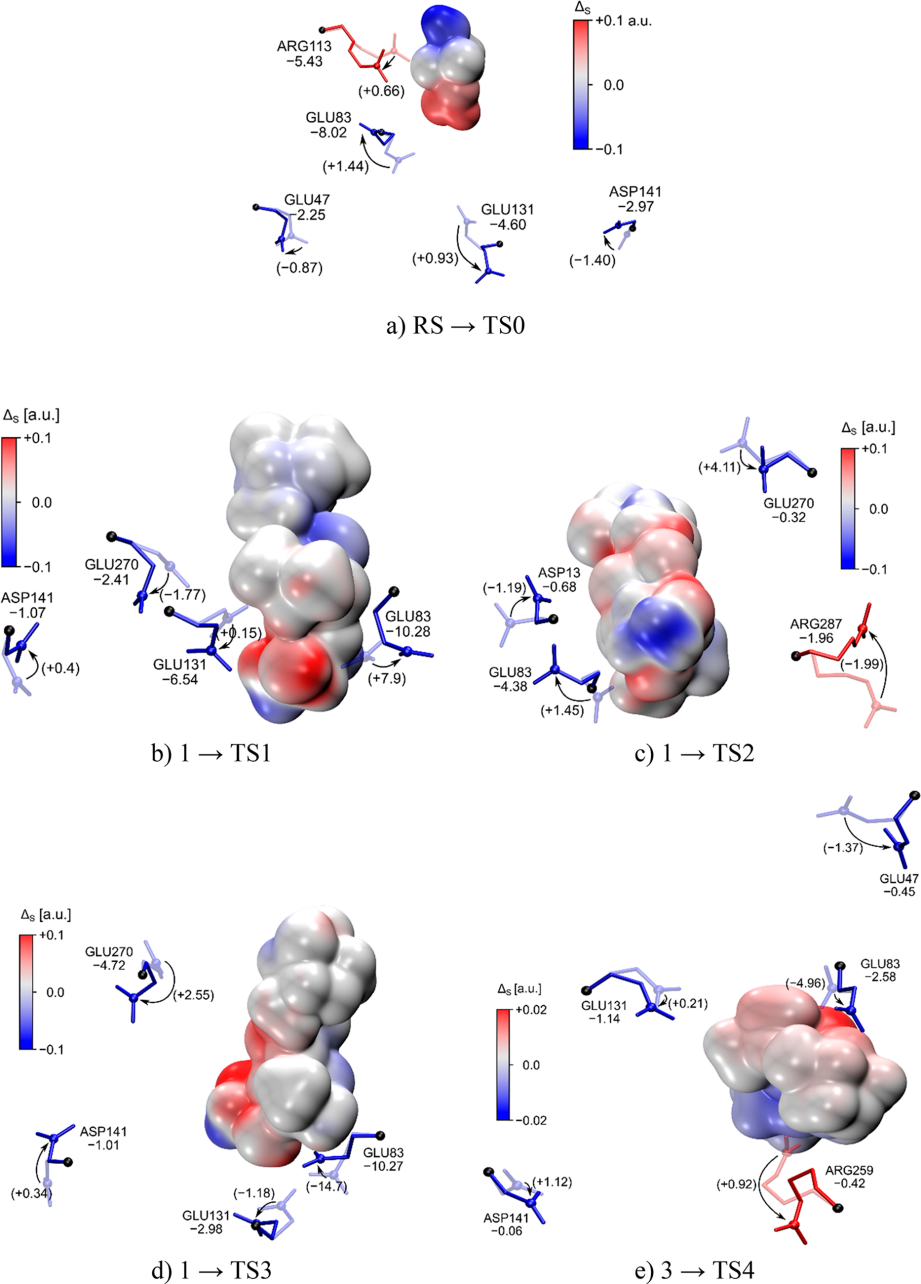
Catalytic fields for all considered reaction mechanisms with conserved residues exerting the most pronounced catalytic activity. 3D view of the surface colored with the Δ_S_ value. The blue color indicates the region where a positively charged catalyst will be beneficial. Red denotes locations beneficial for negatively charged residues. Numerical values in kilocalories per mole denote the lowering of activation energy (DTSS) obtained from the MULTISCAN procedure (conformation denoted by solid color), and the values in parentheses represent the DTSS changes resulting from the use of crystallographic side-chain conformations (faded) and neglecting interactions with remaining charged side chains.

To consider possible conformation changes of side chains, we have scanned the library of CAMMs for amino acid rotamers^34^ using the MULTISCAN procedure described in more detail elsewhere.^21^ This procedure, besides considering the DTSS, also takes into account the relatively strong interactions between charged residues responsible for the nonadditive effects of amino acid multiple mutations.^21^ The inclusion of interactions between charged side chains in the case of the ATP aminoacylation reaction (Scheme 1a) resulted in a total DTSS change reaching up to 2 kcal/mol, and it resulted in a total DTSS change reaching up to 21.5 kcal/mol for tRNA charging reaction (Scheme 1b) when compared with the DTSS values obtained with side-chain conformations from the crystal structure excluding interactions between charged amino acid residues. The corresponding side-chain conformation changes are shown in Figure 5a–e.

The TS structure obtained from the quantum-chemical calculation is definite for a particular protein structure and could be regarded as rigid, so it is computationally much less demanding to determine AA rotamers interacting strongly with the TS and each other. This way, one may single out specific AA side-chain deformations accessible within zero-point vibrations, which could contribute to the lowering of the activation barrier and precisely define the active-site preorganization at the atomic level. Using conventional QM or ONIOM methods, one would have to consider a much larger number of degrees of freedom to obtain such structural data.

It is remarkable that the lowering of the activation barrier for TS3, reaching −14.7 kcal/mol in the case of the Glu83 side chain, seems to play a much more important role in the active-site preorganization than it does for other residues. The inclusion of interactions between charged residues with the MULTISCAN approach has been crucial to achieving agreement with experimental catalytic activities for a series of mutated Kemp eliminases obtained by direct evolution experiments, improving the catalytic activity of the corresponding theozymes.^21^

DTSS contributions arising from the presence of each conserved amino acid (aa) contribute to the crude estimation of the activation energy (eq 4)
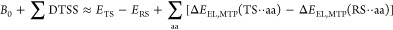
4where *E*_TS_ and *E*_RS_ denote the energies of the models of the transition state (TS) and the substrate (RS), respectively. *B*_0_ has been calculated as the difference in TS and RS energies for the smaller models of reactants shown in Figures 3 and 4. As in Table 1, only the conserved amino acids have been considered, so the *B*_0_ + ∑DTSS does not pretend to be close to the accurate activation energy but certainly indicates major catalytic residues among the nine considered conserved charged residues. The activation barrier change DTSS values presented in Table 1 indicate that for the ATP aminoacylation reaction (Scheme 1a), five residues, Glu47, Glu83, Arg113, Glu131, and Asp141, exert catalytic activity out of all nine conserved charged amino acids. Of course, many more neutral or charged nonconserved residues may contribute to the DTSS, but because of the little similarity between HisRSs, their contributions will be more random in contrast with those of conserved residues. In the case of the second reaction (Scheme 1b), where three different mechanisms^23^ have been considered, the lowest activation barrier, *B*_0_ + ∑DTSS, seems to be associated with the pathway involving TS3, in agreement with Liu and Gauld,^23^ although according to the ∑DTSS value, the path involving TS1 seems to be considerably reduced by interactions with the same conserved residues, as in the case of TS3. The barrier related to TS4 following TS3 via intermediate 3 practically disappears, making TS3 the lowest reaction path. In the TS3 and TS1 cases, tRNA charging is catalyzed by four residues, Glu83, Glu131, Asp141, and Glu270. Although the mechanism involving TS1 has the largest activation barrier reduction, ∑DTSS of 11.48 kcal/mol, the corresponding barrier, *B*_0_, for bare reactants of 84.00 kcal/mol still remains significant.

**Table 1:** Contributions of Conserved Residues from the HisRS Family to Differential Transition State Stabilization (DTSS) (kcal/mol) from the MULTISCAN Procedure^a^

	His + ATP (Scheme 1a)	His-AMP + tRNA (Scheme 1b)			
residue^b^	RS → TS0	1 → TS1	1 → TS2	1 → TS3	3 → TS4
Asp13 (*R* ≈ 22 Å)	+0.57 (+0.30)	+0.83 (+0.52)	–0.68 (+0.51)	+0.65 (−0.10)	+0.09 (+0.04)
Glu47 (*R* ≈ 18 Å)	–2.25 (−1.38)	+0.09 (−1.62)	+1.39 (−0.15)	+1.89 (+1.06)	–0.45 (+0.92)
Glu83 (*R* ≈ 8 Å)	–8.02 (−9.46)	–10.28 (−18.18)	–4.38 (−5.83)	–10.27 (+4.43)	–2.58 (+2.38)
Arg113 (*R* ≈ 10 Å)	–5.43 (−6.09)	+2.82 (−6.21)	+0.63 (+5.38)	+1.48 (−8.71)	+1.49 (−8.11)
Glu131 (*R* ≈ 14 Å)	–4.60 (−5.53)	–6.54 (−6.69)	+0.21 (−0.56)	–2.98 (−1.80)	–1.14 (−1.35)
Asp141 (*R* ≈ 18 Å)	–2.97 (−1.57)	–1.07 (−1.47)	+1.28 (−0.45)	–1.01 (−1.35)	–0.06 (−1.18)
Arg259 (*R* ≈ 7 Å)	+5.26 (+8.44)	+3.40 (+2.37)	+0.15 (+14.42)	+7.03 (+0.18)	–0.42 (−1.34)
Glu270 (*R* ≈ 10 Å)	+0.39 (+1.12)	–2.41 (−0.64)	–0.32 (−4.43)	–4.72 (−7.27)	+048 (−7.24)
Arg287 (*R* ≈ 13 Å)	+3.26 (+1.77)	+1.67 (−1.13)	–1.96 (+0.03)	+1.35 (−1.00)	+0.21 (−0.93)
DTSS	–14.79	–11.48	–4.96	–6.58	–2.38
*B*	32.88^c^	45.81^d^	34.85^d^	26.10^d^	0.26^d^
*B*_0_ = *E*_TS_ – *E*_RS_	37.28	84.00	103.41	18.95	–0.04
*B*_0_ + ∑DTSS	22.49	72.52	98.45	12.37	–2.42

a Numbers in parentheses correspond to side-chain conformation identical as in crystal structure without considering interactions with other residues.

b *R* indicates the distance between Cα and phosphorus atom in angstroms.

c Activation barriers, *B*, reported in the literature: ONIOM study^22^ including Arg113 and Arg259.

d Activation barriers, *B*, reported in the literature: QM/MM study^23^ including Glu83, Arg113, Gln127, and Arg259.

This study allowed us to analyze on equal footing both half-reactions shown in Scheme 1 catalyzed by HisRS. Glu83, Glu131, and Asp141 exert catalytic activity for both half-reactions, whereas Glu47 and Arg113 are specific for ATP aminoacylation, proceeding over TS0 and Glu270 for tRNA charging involving TS3.

**Figure 6:**
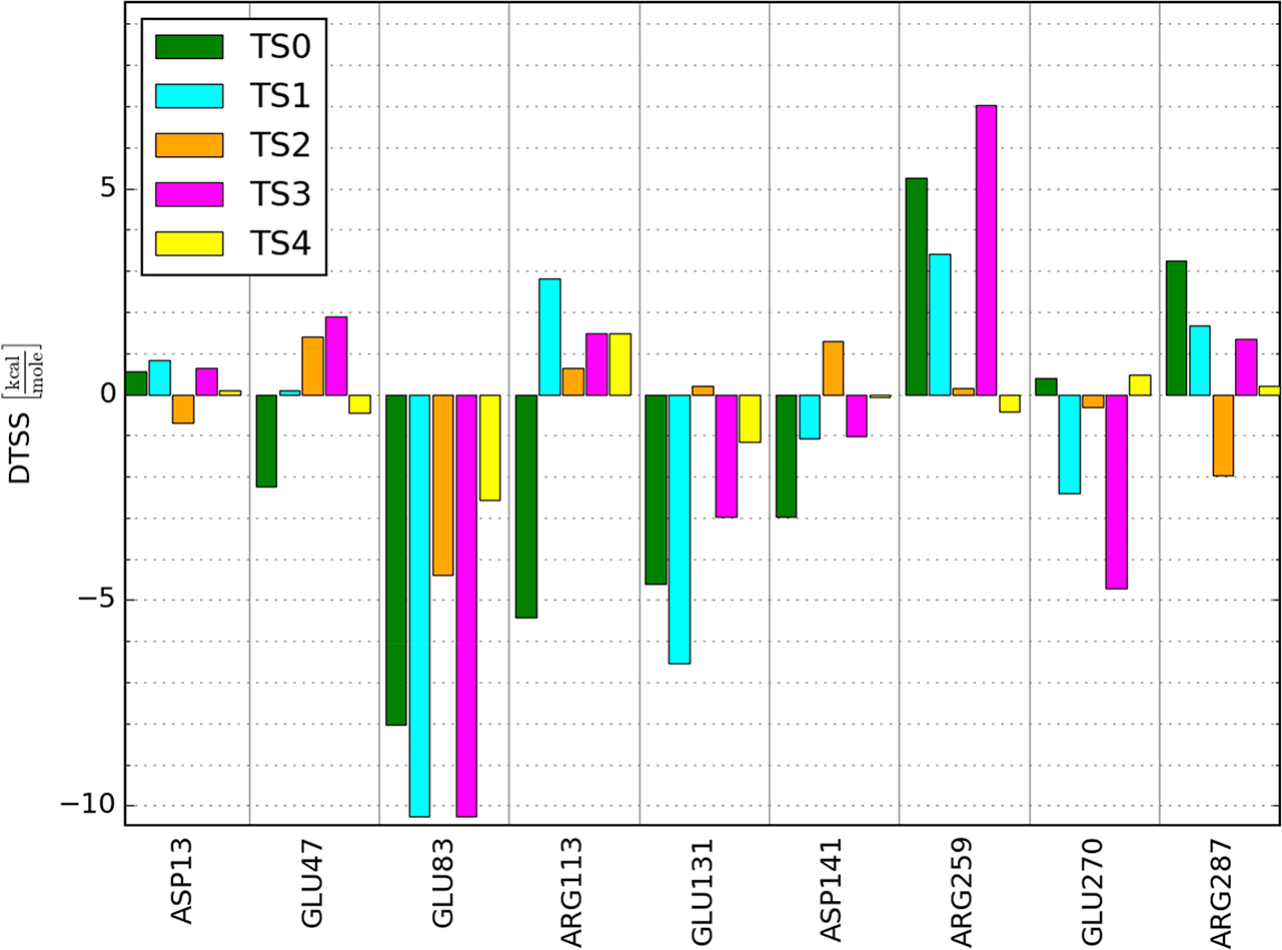
Changes in activation barrier DTSS yielded by the MULTISCAN procedure for conserved charged residues for both reactions and mechanisms catalyzed by HisRS involving transition states TS0, TS1, TS2, TS3, and TS4.

Figure 6 collects the activation barrier changes for all considered reactions, demonstrating the significant sensitivity of the DTSS to a specific unique molecular charge redistribution for each reaction step, reflected by the CF derived from the corresponding wave function via CAMMs. This makes the static CF derived from the minimal reactant and transition state wave functions via CAMMs a sensitive tool for pointing out amino acid residues exerting catalytic activity for every reaction step and mechanism variant.

For class II aminoacyl-tRNA synthetases, available experimental data allowing a comparison with the present results are much more scarce, in contrast with class I aaRS’s for which systematic site-directed mutagenesis results are available.^40^ In the case of HisRS, the catalytic roles of E83 and R113 have been proven by the loss of activity when these residues are replaced by alanine in mutated proteins.^41,42^ Experimental data indicate that other residues like R259 also stabilize transition states. This can be illustrated in the TS3 case, where the transition state stabilization amounts to −6.54 kcal/mol, but when substrate destabilization is also taken into account in the DTSS, then the net DTSS result, +7.03 kcal/mol, is positive. In this particular case, this effect is partially compensated by Glu270 contributing DTSS = −4.72 kcal/mol. These two residues bonded by the salt bridge strongly interact with each other by changing side-chain conformations. This can be illustrated by the significant differences between the crystal structure and the conformations optimal for the extreme lowering of the activation barrier, reaching up to 7 kcal/mol. Such structural details could be essential for the complete understanding of enzyme reactions and rational biocatalyst design. Activation barriers for the critical steps for both half-reactions RS → TS0 and I → TS3 estimated from *B*_0_ + ∑DTSS are only in qualitative agreement with previous reports^22,23^ due to the approximations involved in the present study, probably mainly from the neglect of nonconserved residues near the active site and solvent effects.

There are several limitations of the present model based on static CFs:

(1) Only conserved charged residues are considered, which is necessary due to the little similarity between His-tRNA synthetases.
(2) The applicability of the method was only tested for a charged system, where the electrostatic term could be dominant and the contribution of entropy term could be minimal with respect to the enthalpy term. This is not necessarily a limitation that excludes nonpolar reagents. There are experimentally characterized examples of catalysis driven by the electric field for the Diels–Alder reaction^43^ and hydrogen abstraction from methane.^44^ Supplementing the electrostatic model by dispersion functions^35^ could extend the current model’s applicability to noncharged and nonpolar systems.
(3) Solvent effects are neglected.
(4) The use of the smallest possible models of reactants and transition states is another limitation of the method, however the charge redistribution represented by CAMM is only significant for the immediate neighborhood of broken or formed bonds.The validity of the small model has already been tested against a large model including Mg^2+^ ions, water, and four amino acid residues from the active site by Banik and Nandi.^37^ Using as small as possible of a model of the reacting system has the advantage of minimal errors resulting from superimposing SC atomic multipoles on the TS structure. This is supported by the fact that near the attack, the substrate conformation is usually close to the much more rigid transition-state structure.^45^
(5) The model is unable to deal with tunneling effects. However, in the case of transition states bound to the enzyme via hydrogen -bond chains, one may employ dynamic CFs, which may indicate favorable directions of cooperative proton transfer increasing the catalytic activity.^46^
(6) Because of the approximate (but still nonempirical) character of the DTSS estimates using the CAMM atomic multipole expansion, results should only be treated qualitatively. However, the DTSS-CAMM rankings of the catalytic activity for Kemp eliminase theozyme mutants^29^ or chorismate mutase active-site individual residues were in excellent agreement with the experimental free energies of activation and the accurate MP2 reference results,^9^ respectively. Analogous conclusions have been obtained for inhibitors binding several proteins.^35^

## Conclusions

Catalytic fields derived from substrate and transition-state wave functions enable us to determine the optimal charge distribution of the catalytic environment, which may be naturally confronted with the topology of all conserved charged enzyme amino acids. This could be used to point out the most essential catalytic residues for a given reaction step or mechanism variant. The available library of CAMMs for amino acid side-chain rotamers allows for the rapid scanning of the entire conformational space in the search for active-site structures with optimal catalytic activity, allowing us to inspect in atomic detail the electrostatic preorganization supplementing the previously proposed alternative methods.^47,48^ This is in line with recent independent experimental^17,43,44^ and theoretical^8,9,11−20,49,50^ studies indicating the essential role of electric fields and side-chain motions in enzyme catalysis. Because the substrate, transition state, and side-chain atomic multipole representations were derived from corresponding ab initio wave functions, the presented approach is entirely nonempirical (although approximate). In addition, the perturbational methodology applied in our approach allows for the partitioning of the DTSS catalytic activity into well-defined physical components, in contrast with variational ONIOM or QM/MM methods. The approach proposed in this work, while neglecting entropy, could be useful for screening possible reaction variants, which could later be followed by more computationally demanding free-energy calculations for the most promising cases. For this purpose, the molecular mechanics Poisson–Boltzmann surface area (MMPBSA) approach^51^ could be applied. The DTSS results obtained for the Kemp eliminase theozyme mutants using the CAMM and MMPBSA approaches similarly correlate with the experimental activation free energies (correlation coefficient for CAMM of 0.80 and MMPBSA of 0.79).^21^ The DTSS-CAMM results for ketosteroid isomerase mutants also yielded catalytic activities that were well correlated with the experimental free energies of activation (figure 4d in ref (29)). This could indicate considerable enthalpy–entropy compensation observed for protein binding with transition states.^52^

In addition, supplementing this with an analysis of the dynamic CF, defined as the derivative of the static CF, allows us to determine the role of the possible proton dislocations in hydrogen-bond chains^46^ that enhance the catalytic activity, opening the way for rational reverse biocatalyst design at very limited computational cost without resorting to empirical methods.

## Supporting Information Available

List of 12 selected His tRNA synthetase structures, their complete multisequence alignment results, and rmsd’s for superimposed charged terminal groups (PDF)
