# Supplementary material for: Catalytic Fields as a Tool to Analyze Enzyme Reaction Mechanism Variants and Reaction Steps
Source: J Phys Chem B. 2021 Oct 14;125(42):11606–16. doi: 10.1021/acs.jpcb.1c05256 (PMC8558854; doi:10.1021/acs.jpcb.1c05256)
Supplement: Supplementary file 1 — jp1c05256_si_001.pdf [file jp1c05256_si_001.pdf]

Catalytic Fields as a Tool to Analyze Enzyme Reaction Mechanism Variants and Reaction Steps

Paweł Kędzierski, Martyna Moskal, W. Andrzej Sokalski

Department of Chemistry, Wrocław University of Science and Technology

Wyb. Wyspiańskiego 27, 50-370 Wrocław, Poland.

SUPPORTING INFORMATION

LIST OF SELECTED PDB STRUCTURES

PDB ID	Organism short	Source organism name
1KMM	EC	Escherichia coli
1ADY	TT	Thermus thermophilus
1QE0	SA	Staphylococcus aureus
1WU7	TA	Thermoplasma acidophilum
3HRI	TB	Trypanosoma brucei
3HRK	TC	Trypanosoma cruzi
3NET	NS	Nostoc sp. PCC 7120 = FACHB-418
3RAC	AA	Alicyclobacillus acidocaldarius subsp. acidocaldarius DSM 446
4E51	BT	Burkholderia thailandensis E264
4PHC	HS	Homo sapiens
5E3I	AB	Acinetobacter baumannii
6NHI	EM	Elizabethkingia meningoseptica

PDB ID	chain	Alignment numbering 15	49	114	148	169	183	450	461	489
		Literature (PDB) numbers and corresponding residues								
1KMM	C	13 D	47 E	83 E	113 R	131 E	141 D	259 R	270 E	287 R
1ADY	A	11 D	45 E	81 E	112 R	130 E	140 D	259 R	270 E	287 R
1QE0	A	10 D	44 E	80 E	112 R	130 E	140 D	257 R	268 E	288 R
1WU7	A	14 D	48 E	81 E	110 R	128 D	138 D	265 R	276 E	292 R
3HRI	A	58 D	92 E	125 E	155 R	173 D	183 E	313 R	324 E	340 R
3HRK	A	59 D	93 E	126 E	156 R	174 D	184 E	314 R	325 E	341 R
3NET	A	17 E	51 E	98 D	128 R	146 D	158 D	298 R	309 E	326 R
3RAC	A	28 D	62 E	95 E	125 R	149 E	160 D	306 R	317 E	332 R
4E51	A	19 D	53 E	90 E	120 R	138 E	148 D	265 R	276 E	293 R
4PHC	A	64 D	98 E	130 D	157 R	177 D	188 D	326 R	337 E	362 R
5E3I	B	13 D	47 E	85 E	115 R	133 E	143 D	261 R	272 E	289 R
6NHI	A	11 D	45 E	100 D	130 R	148 D	158 E	293 R	304 E	320 R

Complete multi sequence alignment results for 12 His tRNA synthetases

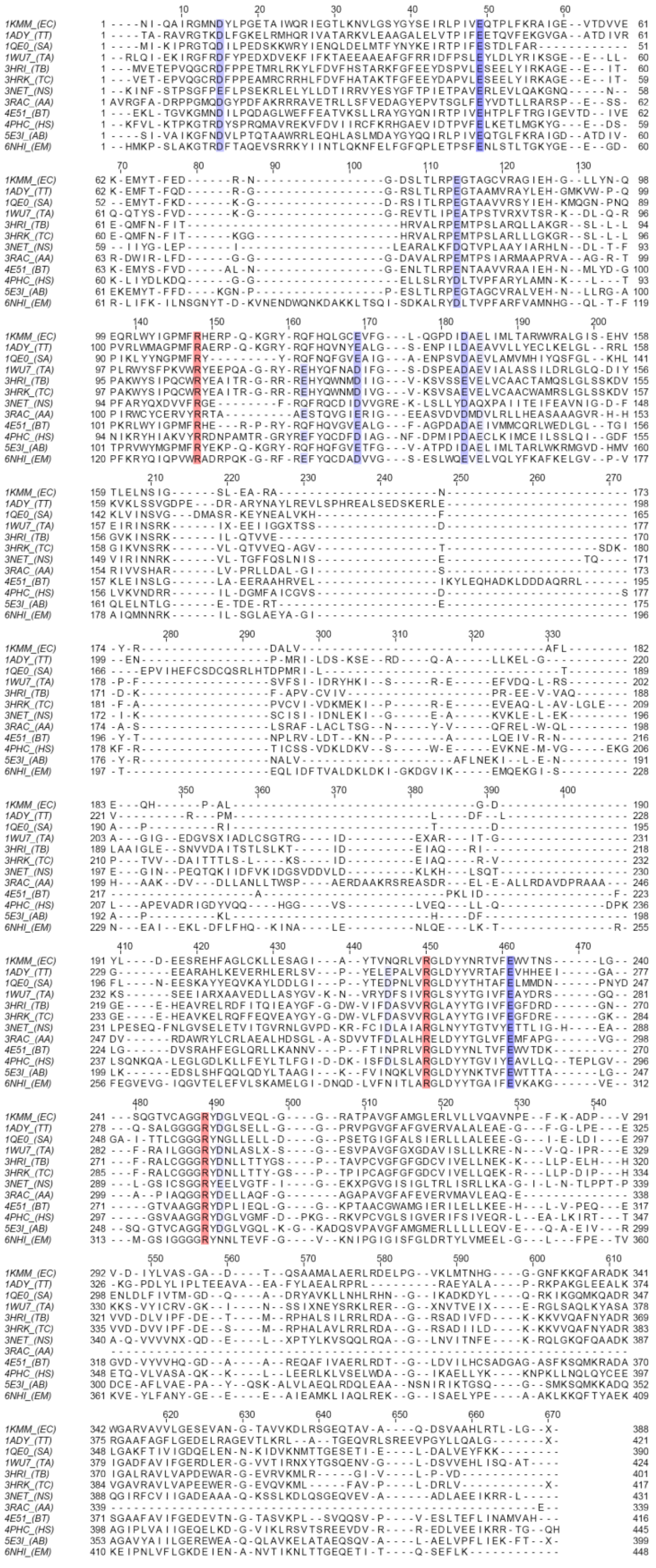

Root mean square deviations RMSD in [Å] for alpha carbon atoms (CA) and charged terminal groups CO_2^- or $C(NH_2)^+$ (CS) for 9 HisRS structures superimposed by alpha carbons of conserved charged residues on 1kmmC

1adyA.pdb -> 1kmmC.pdb: RMSD CA 0.561, RMSD SC 0.852

1qe0A.pdb -> 1kmmC.pdb: RMSD CA 1.431, RMSD SC 1.520

1wu7A.pdb -> 1kmmC.pdb: RMSD CA 1.289, RMSD SC 3.275

3hriA.pdb -> 1kmmC.pdb: RMSD CA 0.928, RMSD SC 3.137

3hrkA.pdb -> 1kmmC.pdb: RMSD CA 0.733, RMSD SC 1.088

3racA.pdb -> 1kmmC.pdb: RMSD CA 1.123, RMSD SC 1.442

4e51A.pdb -> 1kmmC.pdb: RMSD CA 0.375, RMSD SC 0.589

4phcA.pdb -> 1kmmC.pdb: RMSD CA 0.668, RMSD SC 0.976

5e3iB.pdb -> 1kmmC.pdb: RMSD CA 0.295, RMSD SC 0.885

6nhiA.pdb -> 1kmmC.pdb: RMSD CA 1.520, RMSD SC 2.015
